# The mechanisms in glucose metabolism of aging hippocampus

**DOI:** 10.1002/ibra.12201

**Published:** 2025-08-12

**Authors:** Rui He, Fuxing Zhao, Zhiyu Yang, Tingting Wang, Yaohui Zhang, Jinglan Quan, Gaohong Zhu

**Affiliations:** ^1^ Department of Nuclear Medicine The First Affiliated Hospital of Kunming Medical University Kunming China

**Keywords:** aging, ALG5, glucose metabolism, hippocampus, STT3A

## Abstract

With the intensification of the aging society, the incidence of various neurodegenerative diseases is on the rise. The hippocampus is susceptible to age‐related neuronal decline and is the earliest and crucial region affected in the transition from healthy aging to neurodegenerative diseases. Before the diagnosis of neurodegenerative diseases, there is already a decline in brain energy metabolism, with the disruption of energy metabolism serving as the primary mechanism leading to neuronal damage. This triggers complex signaling mechanisms both inside and outside the brain during the aging process. Glucose serves as the primary energy source for brain tissues, and a decrease in glucose metabolism is an early indicator of age‐related functional changes in the brain. Therefore, understanding the pathophysiological basis of glucose metabolism in the aging hippocampus, as well as the underlying mechanisms, is crucial in comprehending cognitive aging. Such understanding is integral for early intervention and the mitigation of memory and learning impairments caused by energy metabolism. In this review, we have delved into the characteristics of energy metabolism, focusing specifically on glucose metabolism, as well as exploring the molecular foundations and associated mechanisms present within hippocampal neuronal cells under both normal and aging conditions. Notably, our investigation has highlighted the vital roles played by ALG5 and STT3A, key molecules involved in N‐glycosylation, in influencing GLUT expression and the rate of membrane transport, regulating glucose metabolism, and thereby influencing cellular glucose uptake. The exploration of this study direction holds considerable promise for future endeavors.

## Graphical abstract

Cognitive decline in elders stems from the irreversible loss and functional decline of neurons, with the hippocampus being particularly susceptible to age‐related neuronal deterioration. Glucose acts as the primary fuel for the brain, and a constant provision of glucose to the hippocampus is essential for memory and cognitive functions. Aging neurons are accompanied by an increase in insulin resistance and a decrease in sensitivity to insulin receptors, such as IR and IGF‐IR, resulting in inefficient glucose utilization. Several signaling pathways in the hippocampus, notably the insulin PI3K/Akt/AS160 pathway, greatly impact glucose metabolism. This pathway predominantly regulates the translocation of GLUT4, which possesses two unique glycosylation sites. When insulin concentrations decrease in vivo, GLUT4 rapidly relocates from GSVs to the plasma membrane, promoting glucose uptake and maintaining blood glucose homeostasis. In this review, we have delved into the characteristics of energy metabolism, focusing specifically on glucose metabolism, as well as exploring the molecular foundations and associated mechanisms present within hippocampal neuronal cells under both normal and aging conditions. Notably, our investigation has highlighted the key molecules involved in N‐glycosylation, in influencing GLUT expression and the rate of membrane transport, regulate glucose metabolism, and thereby influence cellular glucose uptake.

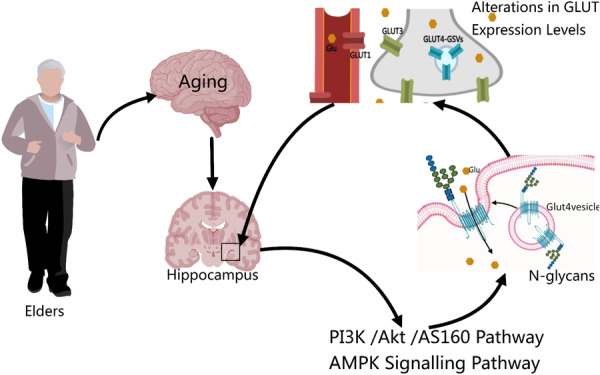

## 1 INTRODUCTION

As the brain experiences a series of molecular and cellular alterations throughout the aging process, it is important to note that approximately 20%–40% of individuals aged between 60 and 78, regardless of their overall health, experience notable declines in cognitive performance across various domains, encompassing working memory, spatial memory, situational memory, and processing speed.^1^ These are mainly responsible for the hippocampus. These declines in cognitive function coincide with the escalating prevalence of neurodegenerative brain disorders like Alzheimer's disease (AD) and Parkinson's disease (PD).^2^, ^3^ Among these, the hippocampus stands as a vulnerable region to age‐related neuronal deterioration, representing a critical site in the transition from normal aging to the onset of neurodegeneration.^4^, ^5^ Brain energy metabolism undergoes subtle modifications during the natural progression of aging. Glucose metabolism imaging reveals that the hippocampus exhibits significantly different glucose metabolism during normal aging compared to AD patients.^6^ These transformations often emerge in conjunction with various energy metabolism‐related pathways in brain cells, including glucose transport, mitochondrial electron transport, DNA repair, and neurotrophic factor signaling.^7^ It is worth noting that disruptions in energy metabolism serve as a prominent mechanism responsible for neuronal damage, with glucose metabolism holding potential as an indicator of these age‐related pathologies.^8^, ^9^

Glucose, functioning as the primary source of energy, stands as nearly the exclusive supplier of energy for brain tissue. Consequently, changes in glucose metabolism levels accompany the physiological activities and pathological processes within the brain. Substantial research has shed light on the fact that age‐related decline in brain energy metabolism pertains to glucose, manifested in the hippocampus as physiological changes closely related to cognition.^6^, ^10^, ^11^ While age‐related metabolic decline may be perceived as a “dysfunction,” it could potentially represent an evolutionary adaptation where the hippocampus undergoes adaptive alterations in glucose utilization during aging and specific pathological states. The modulation of glucose utilization capacity assumes an essential role in safeguarding hippocampus from potential self‐inflicted harm.

The investigation of the effects of aging on organ function plays a pivotal role in the field of neuroscience. Acquiring knowledge about the alterations in glucose metabolism and the underlying mechanisms in the aging hippocampus is essential for implementing early interventions and mitigating the learning and memory impairments induced by declining energy metabolism. In this comprehensive review, we aim to provide a concise overview of the characteristics of glucose energy metabolism and the molecular foundations in hippocampal neuronal cells under normal and aging conditions. This contributes to the advancement of our understanding of the interplay between hippocampal glucose metabolism and age‐related neurodegenerative diseases.

## 2 ENERGY METABOLISM IN NORMAL AND AGING HIPPOCAMPUS

### 2.1 Energy metabolism in the normal brain

Brain is most active organ when it comes to energy metabolism, accounting for approximately 20% of the body's overall energy consumption despite weighing a mere 2.5% of the total body weight.^12^ Consequently, neurons, the primary cellular constituents of the brain, utilize approximately 70%–80% of the total energy, while the remaining portion is allocated to glial cells such as astrocytes, oligodendrocytes, and microglia.^13^ Glucose conventionally serves as the predominant energy source for the central nervous system (CNS),^14^ though alternative substrates such as ketone bodies,^15^ lactate,^16^ and others may be utilized to a lesser extent. Within the confines of normal physiological conditions, glucose traverses the blood‐brain barrier (BBB), gaining access to the brain, and subsequently crosses cell membranes to enter the cytoplasm. Once there, it undergoes a series of transformations, generating pyruvate, which enters the mitochondria and assimilates into the tricarboxylic acid cycle through oxidative decarboxylation. As a result, adenosine triphosphate (ATP) is released as a means of providing the vital energy supply.^17^ It is crucial to note that any dysregulation in glucose metabolism within the brain can yield an array of detrimental consequences, potentially serving as the causative factor for neuronal damage and a variety of brain‐related pathologies.

### 2.2 Energy metabolism in the normal hippocampus

The hippocampus, renowned for its significance in systemic aging and lifespan regulation, demonstrates a distinctive developmental trajectory characterized by an inverted U‐shape, reaching maturity typically around the age of 50.^9^ As the paramount region responsible for memory and cognitive functions, the hippocampus assumes the role of the cerebral memory hub, chiefly overseeing episodic memory,^18^ spatial navigation,^19^ cognition,^20^ and stress responsiveness.^21^ Glucose, serving as the primary energy source for the brain, assumes a critical role in fostering and preserving learning and memory functions. Extensive studies conducted on humans^22^, ^23^ and animals^24^, ^25^ alike have consistently revealed glucose to be a principal regulator of cognitive capacities and memory functions within the brain. Numerous animal and population studies have provided confirmation of the influence of glucose on learning and memory functions, and the prominent theories currently postulated include the hippocampal hypothesis,^26^, ^27^ the insulin hypothesis,^28^, ^29^ acetylcholine synthesis,^30^, ^31^ K‐ATP channels,^32^ and others. In conclusion, sustained energy metabolism within the hippocampus constitutes the linchpin for preserving the brain's memory and cognitive functions, necessitating further comprehensive investigation into its specific mechanisms.

### 2.3 Energy metabolism in the aging hippocampus

A substantial proportion of individuals aged 60–78 (around 20%–40%) encounter notable declines in multiple cognitive domains, encompassing working memory, spatial awareness, episodic memory, and processing speed.^1^, ^33^ These cognitive alterations mainly occur in the hippocampus and are linked to an array of factors, encompassing neuroanatomical changes,^34^ the uneven thinning of cortical volume,^35^ reduction in gray and white matter,^36^ diminished metabolism and energy utilization,^37^ among others. Disorders of energy metabolism are thought to be the main mechanism of neurological injury, and in the hippocampus, they are important factors in cognitive dysfunction and related pathological transformations. Disruptions in energy metabolism induced by glucose disturbances of hippocampal neurons inevitably influence the normal functioning.^38^, ^39^ Functional neuroimaging assessment showed that glucose hypometabolism and mitochondrial dysfunction were early indicators of age‐related functional transitions in hippocampal neurons.^39^ Studies employing positron emission tomography have consistently shown age‐associated reductions in hippocampal regions' glucose utilization across subjects of varying ages.^40^, ^41^ These findings underscore the role of hypometabolism in cognitive deterioration in the aging hippocampus. It is increasingly evident that alterations in bioenergetics initiate a complex cascade of internal and external signaling mechanisms during the hippocampal aging process. These mechanisms coordinate the adaptive responses of multiple hippocampal cells to optimize the function and safeguard against injury and disease. Once an imbalance in hippocampal energy metabolism arises, resulting in the activation of transcription factors at the molecular level, the expression of relevant proteins is induced, altering the resilience of hippocampal cells to various stresses and ultimately leading to the onset of diverse disorders.^7^

## 3 GLUCOSE METABOLISM IN NORMAL AND AGING HIPPOCAMPUS

Glucose, the primary energy source for brain tissue, undergoes distinct metabolic pathways depending on the cell type and the selective expression of metabolic enzymes. Neurons predominantly rely on oxidative metabolism, while astrocytes mainly utilize glycolysis.^42^, ^43^ Besides ATP production, glucose is utilized to generate metabolic intermediates essential for synthesizing fatty acids, membranes, and other lipids crucial for phospholipid formation.^44^ It also provides amino acids for protein synthesis and neurotransmitter production,^45^ 5‐carbon sugars for nucleotide synthesis,^46^ and serves as a precursor for glycogen synthesis in astrocytes. Disruptions in glucose transport or utilization within the CNS can negatively impact neuronal activity, particularly in hippocampal regions.^37^ Reduced glucose utilization is observed in AD, and cognitive deficits associated with AD can be ameliorated by glucose administration^47^ and insulin therapy.^48^ Studies have shown enhanced glucose metabolism in the cerebral cortex upon low‐dose insulin administration^49^ and demonstrated changes in brain organ glucose metabolism between young and aged mice, indicating a gradual decline in insulin sensitivity in certain brain regions as individuals age.^9^ This decline in insulin sensitivity may contribute to age‐related brain diseases like AD and PD. Early insulin abnormalities may contribute to the pathological cascade of AD before the manifestation of clinical dementia symptoms.^50^

## 4 MOLECULAR BASIS OF GLUCOSE METABOLISM IN AGING HIPPOCAMPUS

Glucose serves as the primary source of energy for the brain, and the decline in glucose uptake and metabolism is a significant characteristic of brain aging.^51^, ^52^ Understanding the alterations in glucose metabolism and their impact on neuronal activity is crucial for early intervention and delaying cognitive impairments caused by energy metabolism.

### 4.1 Facilitative glucose transporters (GLUTs)

The uptake of glucose by the brain is not influenced by peripheral glucose levels, but rather by the energy demands of active neurons. Neurons themselves lack the ability to store or synthesize glucose and thus rely on a continuous supply from the extracellular environment.^53^ The progressive decline in glucose uptake and metabolism leads to a persistent energy deficit in hippocampal cells, resulting in impaired cellular function. The transmembrane transport of glucose relies on the coordinated action of various glucose transport proteins. In mammals, GLUTs can be categorized into two main groups: sodium‐glucose co‐transporters (SGLT) and GLUTs.^54^ GLUTs are transmembrane glycoproteins, responsible for mediating glucose transport into and out of the cell. They function as unidirectional transporters that selectively bind to glucose molecules, allowing them to cross the membrane along concentration gradients. Consequently, the transport of glucose through GLUTs is widely acknowledged as the rate‐limiting step in the cell's high‐energy metabolism.^55^

There are 14 types of GLUTs identified in human tissues, including GLUT1 to GLUT14. These GLUTs share common structural features, including twelve transmembrane helices that form the N‐terminal and C‐terminal domains, seven conserved amino acid residues in each domain, a single N‐glycosylation site in the extracellular loop between the first and second transmembrane domains, and a structural domain consisting of four α‐helices in the intracellular soluble region. The intracellular soluble region also contains another structural domain with four α‐helices. Additionally, certain amino acid residues, such as Asp, Glu, Ser, and Thr, found in other transmembrane regions, are believed to play a role in glucose binding and facilitate its passage through the hydrophilic core of GLUTs.^56^, ^57^, ^58^, ^59^, ^60^ Studies have revealed that the human brain expresses more than ten GLUTs, including GLUT1 to GLUT8, GLUT9, GLUT12, and GLUT13.^56^, ^57^ Among these GLUTs, GLUT1 to GLUT4 have been extensively studied as fundamental GLUTs.

It is crucial to gain a comprehensive understanding of glucose metabolism in the context of aging hippocampus, investigate how alterations in GLUTs expression and function impact glucose transport in different regions of the hippocampus, and their association with cognitive function.

#### 4.1.1 GLUT1

GLUT1, a protein consisting of 492 amino acids,^61^ is encoded by the solute carrier family 2 member 1 (SLC2A1) gene situated on the short arm of chromosome 1. It exists in two isoforms, a highly glycosylated 55 kDa form and a lowly glycosylated 45 kDa form. The former is predominantly expressed in the cerebral microvascular endothelium, facilitating glucose transportation across the BBB, while the latter is found in all other neuronal cells and distributed in the choroidal and ventricular epithelial cells. GLUT1 is abundant on endothelial cells lining the walls of small blood vessels, making it a crucial model for solute transport.^62^ In the CNS, glucose, the primary energy source, can either diffuse directly into neuronal cells through the extracellular space or rely on GLUT1 on the membranes of microvascular endothelial cells for transport into astrocytes.^63^, ^64^, ^65^ GLUT1 expression is influenced by various factors, including estrogen, asphyxia, inhibition of oxidative phosphorylation, mitochondrial function, ATP levels, partial pressure of oxygen, surrounding sugar concentrations, and regulation by genes at different stages of the process. Additionally, different diseases, such as neurodegenerative disorders, stroke, and traumatic brain injury, can affect GLUT1 expression.^66^ In turn, the affected GLUT1 expression can further advance neurodegenerative disorders. A study by Zlokovic's team using transgenic mice demonstrated that GLUT1 deficiency from 2 weeks of age resulted in reduced brain glucose uptake and neuronal dysfunction at 6 months of age.^67^ On the other hand, GLUT1 deficiency in endothelial cells led to the disruption of the BBB, which has been identified as a contributing factor in the pathogenesis of AD.^67^ In contrast, direct injection of adeno‐associated viral vectors containing the human GLUT1 promoter into the brains of GLUT1‐deficient mice led to GLUT1 expression in cortical limbic regions, increased cerebrospinal fluid glucose levels, and improved motor function.^68^ Therefore, GLUT1 deficiency causes early brain damage.

#### 4.1.2 GLUT3

GLUT3, comprised of 496 amino acids with a molecular weight of 54 kDa, is encoded by the solute carrier family 2 member 3 (SLC2A3) gene located on chromosome 12p13.31.^69^ GLUT3 exhibits a high affinity (Km = 1.4) for its primary substrate, d‐glucose. This attribute facilitates neuronal glucose uptake, ensuring a constant supply of glucose to neurons, which is imperative for regulating neuronal metabolism and generating the energy required for cognitive function. In the brain, GLUT3 is predominantly distributed in the hippocampal CA1 and CA3 regions, as well as the pyriform cortex.^70^, ^71^ Additionally, studies conducted on human brain tissue indicate that GLUT3 is primarily localized in axonal and dendritic processes within neocortical and deep cortical structures.^72^ Synaptic activity, various metabolic, hormonal, pharmacological, and toxic factors can induce GLUT3 expression.^73^, ^74^ The regulation of GLUT3 expression allows for effective upregulation or downregulation depending on specific conditions and the subsequent energy demands. GLUT3 deficiency can lead to neuronal energy failure, thereby impairing neuronal and/or brain development and function.^75^ Studies have demonstrated reduced GLUT3 expression in the spinal cord and brain of aged rats.^76^ Notably, GLUT3 responsiveness declines with age in the hippocampus and cerebellum.^77^ Reduced neuronal glucose uptake mediated by GLUT3 is a common characteristic of neurodegenerative diseases.^78^, ^79^ A growing body of evidence suggests a connection between impaired glucose metabolism in the brain and AD, which is associated with Tau hyperphosphorylation and the accumulation of neurofibrillary tangles due to reduced levels of GLUT1 and GLUT3.^38^, ^80^ Other studies indicate that decreased GLUT3 levels are linked to more severe AD pathology.^81^ These precedent studies strongly emphasize the vital role of GLUT3 in maintaining the physiological function of brain neurons, particularly in relation to hippocampal ATP‐dependent axonal transport processes, synaptic plasticity, and aging.

#### 4.1.3 GLUT4

GLUT4, composed of 509 amino acids with a molecular weight of 54,787 Da, is encoded by the solute carrier family 2 member 4 (SLC2A4) gene located on chromosome 17p13.1. In contrast to GLUT1 and GLUT3, GLUT4 is a glucose transporter that relies on insulin for its activity.^82^ GLUT4 is expressed in various regions of the brain, including the hypothalamus, motor sensory cortex, pituitary gland, and hippocampus.^83^ Within the hippocampus, GLUT4 expression is localized to the pyramidal cell layer in the CA1 region and the granule cell layer of the dentate gyrus in the CA3 region.^84^ Interestingly, GLUT4 can be found throughout neurons, including nerve endings.^84^ Structural studies have demonstrated that GLUT4 possesses a distinct glycosylation site in the extracellular helical transmembrane segment and a unique intracellular helical residue (ICH5), which regulate endocytosis and exdocytosis, playing a role in insulin signaling and membrane trafficking.^84^, ^85^, ^86^, ^87^, ^88^ Under normal physiological conditions, over 90% of GLUT4 is located within intracellular vesicle‐like structures referred to as GLUT4 storage vesicles (GSVs), such as Golgi, microsomes, and tubular vesicles. Upon insulin stimulation, GLUT4 rapidly translocates from GSVs to the plasma membrane, allowing for increased glucose uptake into the cytosol. When insulin stimulation ceases, GLUT4 is transported back from the plasma membrane to the intracellular space through endocytosis and stored once again in GSVs. This dynamic process is the main mechanism behind the insulin‐stimulated increase in glucose uptake observed in insulin‐sensitive tissues and plays a crucial role in glucose homeostasis.^89^ Studies have demonstrated that during action potential firing, nerve endings rely on GLUT4 as a regulatory system for glycolysis to meet the increased energy demands driven by neuronal activity. Furthermore, synaptic activity triggers the insertion of GLUT4 into the axon plasma membrane, further supporting its role in neuronal glucose uptake.^84^ The hippocampus, responsible for the integration of learning and memory formation, requires substantial energy supply. Insulin‐dependent GLUT4 plays a pivotal role in providing glucose during hippocampus‐dependent learning and memory processes. Inhibition of GLUT4‐mediated glucose uptake in hippocampal neurons has been shown to impair memory acquisition, underscoring the significance of GLUT4 as a key regulator of hippocampal memory processing.^90^ The translocation of GLUT4 serves as a mechanism to provide increased metabolic. During mental load tasks or memory formation that sustain neuronal activity, GLUT 4 levels are elevated in the brain to meet metabolic demands.^91^ Specifically, it translocates to the plasma membrane in response to insulin stimulation, thereby enhancing glucose uptake in the hippocampus and potentially improving hippocampus‐dependent cognitive function.^92^ Furthermore, reduced expression of GLUT4 was observed in both the cortex and hippocampus of AD models.^93^ Neurotoxic proteins, such as Aβ, generated in AD were also found to inhibit GLUT4 and phosphofructokinase, ultimately impeding glucose uptake, aerobic glycolysis, and ATP synthesis, thereby compromising ATP production.^94^

**Figure 1:**
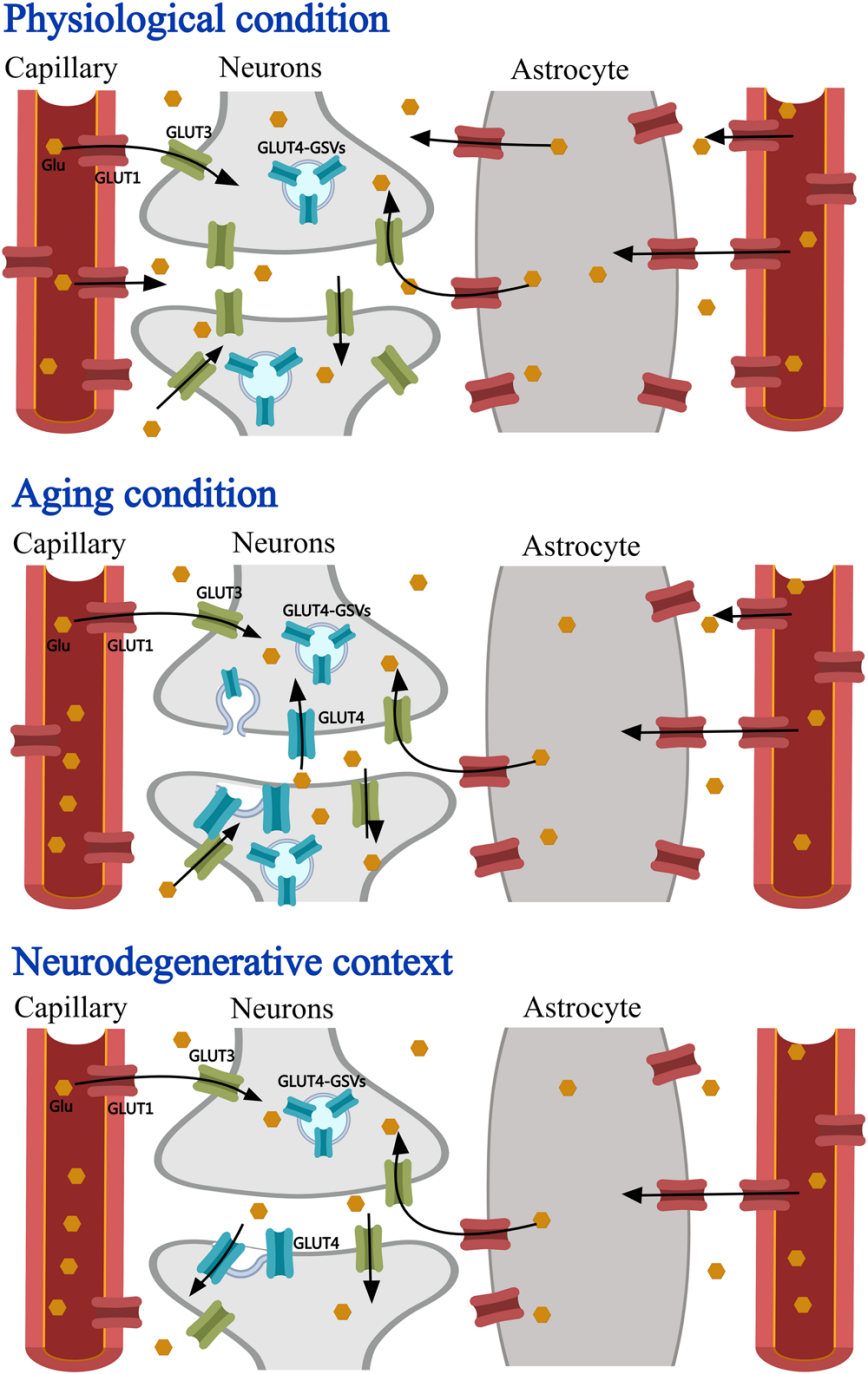
Alterations in GLUT expression levels in different states. Under physiological conditions, glucose is the primary energy source for the central nervous system (CNS). Its entry into the intercellular fluid through the blood‐brain barrier (BBB) relies on GLUT1, while neurons in the brain depend on GLUT3 as the principal glucose transporter to ensure a stable glucose supply. Additionally, GLUT4, an insulin‐responsive glucose transporter, functions as a “reserve” glucose supply pathway within neurons and is typically stored in vesicles. During cognitively demanding tasks, memory formation, and other activities that require sustained neuronal activity, the metabolic demands on the brain increase, leading to the translocation of GLUT4 vesicles in response. Neuronal energy deficiency is a typical manifestation of brain aging, and the process may be associated with diminished levels of both GLUT1 and GLUT3. A corresponding increase in GLUT4 translocation occurs with increased insulin. And increasing insulin along with insulin resistance, impaired transport of GLUT4, and lower levels of GLUT1 and GLUT3, lead to persistent shortage of brain energy supply, brain cell dysfunction, and accumulation of neurotoxic proteins, and eventually cause cognitive decline and neuropsychiatric symptoms, and even neurodegenerative diseases. GLUT, glucose transporters; GSVs, GLUT4 storage vesicles. [Color figure can be viewed at wileyonlinelibrary.com]

To summarize, glucose serves as a crucial energy source within the CNS, where it is transported by GLUT1 from brain microvascular endothelial cells to astrocytes or intercellular fluid. Meanwhile, GLUT3 plays a pivotal role in supplying glucose to stabilize neurons in the brain, particularly in the hippocampus, and may also be implicated in the aging process. Moreover, within the hippocampus, GLUT4, an insulin‐dependent glucose transporter, acts as a specialized conduit for additional glucose supply and serves as a “reserve” channel in hippocampal neurons. It is believed that this mechanism plays a significant role in the proper functioning, metabolism, and aging of hippocampal neurons. The alterations in GLUT levels at different states are illustrated in Figure 1.

### 4.2 Insulin receptor family

The hippocampus is particularly vulnerable to disruptions in glucose levels, making it highly sensitive to insulin deficiency. Insufficient insulin compromises the hippocampus' ability to form new memories and leads to its gradual degeneration and neuronal death over time. Insulin receptors (IR) play a crucial role in insulin signaling, facilitating the activation of insulin receptor substrates (IRS) that can traverse the BBB and be internalized by neurons, enabling the imaging of glucose utilization.

The IR family belongs to the receptor tyrosine kinase (RTK) subfamily, characterized by heavily glycosylated transmembrane proteins consisting of various structural subunits. These include the IR, insulin‐like growth factor‐1 receptor (IGF‐1R), and insulin receptor‐related receptor (IRR). Members of the IR family form heterotetramers comprised of two α‐subunits and two β‐subunits connected by disulfide bonds. The α‐subunit serves as the ligand‐binding site, while the β‐subunit represents the intracellular segment containing the tyrosine kinase active region. Notably, IRR is often considered an “orphan” receptor due to the absence of identified endogenous ligands.^95^

#### 4.2.1 IR

The IR consists of two repetitive extracellular domains (ECDs), a transmembrane helix, and two intracellular cytoplasmic domains containing tyrosine kinase structural domains (TKDs).^96^ The α‐subunit is located entirely outside the cell membrane and contains the insulin‐binding site. The β‐subunit cytoplasmic part possesses insulin‐regulated receptor tyrosine kinase catalytic activity.^97^ The IR is not only widely distributed in peripheral tissues but is also expressed in glial cells and neurons, including the cerebral cortex, hypothalamus, limbic system, hippocampus, olfactory bulb, cerebellum, and cerebral microvascular endothelial cells.^98^ In the CNS, IR and its downstream cell signaling pathways play a role in modulating neuronal function and activity, potentially influencing learning and memory processes.^99^ Subsequent investigations have revealed reduced expression and activation of IR and IGF‐1R in the brains of AD patients compared to healthy individuals.^100^ Collectively, these studies suggest a close association between insulin resistance and IR in the CNS, making it an important target for future AD treatment approaches.

#### 4.2.2 IGF‐1R

The α‐subunit of the IGF‐1R encompasses a ligand‐binding region rich in cysteine residues. This region exhibits the highest affinity for insulin‐like growth factor‐1 (IGF‐1) and the lowest affinity for insulin. The ligand‐binding capacity of this region is closely associated with its biological function. The β‐subunit of IGF‐1R contains binding sites for IRS, ATP, and tyrosine kinase regions, displaying over 80% homology with the IR.^101^, ^102^ The expression of IGF‐1R is widely observed in the brain, particularly in neuronal cells. IGF‐1, the principal ligand of IGF‐1R, exerts insulin‐like effects by promoting glucose uptake in tissues, stimulating gluconeogenesis and glycolysis, enhancing amino acid and glycogen uptake, and consequently promoting protein and lipid synthesis.^103^ Studies have demonstrated aberrant IGF‐1R signaling in the nervous system, leading to reduced and abnormal neuronal cell numbers, impaired myelin formation, and increased apoptosis.^104^ Specific blockade of IGF‐1R in the rat choroid plexus has also resulted in cerebral amyloidosis, cognitive deficits, and hyperphosphorylated tau deposition resembling AD.^105^ Furthermore, in‐depth analysis of the IGF‐1 and IGF‐1R genes in German‐Jewish centenarians, their offspring, and matched controls revealed a significant number of heterozygous mutations in the IGF‐1R gene of centenarians. These mutations altered the IGF signaling pathway and increased susceptibility to human longevity, suggesting a central role for the IGF‐1R axis in the aging process.^106^

To summarize, the uptake of glucose by hippocampal neurons heavily relies on insulin levels. Any decrease in the sensitivity of IR, expression, and activation of IGF‐1R, as well as disruptions in their signaling pathways, can significantly impact the binding of insulin and IGF1. Consequently, the efficiency of glucose utilization in hippocampal neurons becomes compromised, leading to varying degrees of disruption in neuronal function, synaptic plasticity, and cellular integrity. This highlights the potential central importance of IR, IGF‐1R, and their signaling pathways in regulating glucose uptake during the aging process.

### 4.3 N‐glycosylation

Glycosylation, a crucial co‐translational and posttranslational modification of proteins and lipids, plays a significant role in regulating their function, stability, and dynamics.^107^, ^108^, ^109^ Among these modifications, N‐glycosylation is one of the most prevalent alterations found in secreted proteins, especially in the CNS.^110^, ^111^, ^112^ The onset of N‐glycosylation occurs primarily in the endoplasmic reticulum and Golgi apparatus of dendrites and cell bodies of hippocampal neurons.^113^ Notably, research suggests that certain glycan epitopes play critical roles in brain development and function, and abnormal glycosylation has the potential to be a therapeutic target for congenital disorders and cancer.^114^, ^115^ Furthermore, blocking the maturation of neuronal N‐glycans has been linked to neurodegenerative phenotypes.^116^ Studies have shown that blocking N‐glycosylation of membrane receptors and neurotrophic factors in hippocampal dendrites disrupts the consolidation of peripheral fear memories, emphasizing the importance of identifying key target proteins of N‐glycosylation in the hippocampus.^117^ Moreover, pharmacological inhibition of N‐glycosylation has demonstrated its essential role in maintaining long‐term enhancement in hippocampal CA1 neurons.^118^ However, the precise mechanisms by which N‐glycosylation maintains hippocampal physiological function remain largely unknown and require further extensive experimental investigations.

#### 4.3.1 N‐glycosyl chains in GLUTs

GLUTs possess a conserved N‐glycosylation site (Asn‐Xaa‐Thr/Ser) in the extracellular loop between transmembrane domains 1‐2. Interestingly, GLUT4 stands out by having an additional sugar group attached to its extracellular helical transmembrane segment, the Asn57 site, distinguishing it from other GLUT isoforms. N‐glycosylation is commonly associated with the recognition of proteins and plays a critical role in their proper folding, stability, classification, and overall functionality.^119^ Notably, the molecular sizes and mobility of GLUT1 expressed in cerebral capillaries, where the BBB is present, have been identified to differ on sodium dodecyl sulfate–polyacrylamide gel electrophoresis. The variances in apparent molecular weights observed in transporters within the BBB, human erythrocytes, and other cells can be attributed to variations in the extent of N‐glycosylation.^120^, ^121^ The glycosylation of GLUTs significantly contributes to their ability to perceive changes in the cellular microenvironment and regulate glucose homeostasis. Exploring whether the unique dual glycosylation sites of GLUT4 are linked to its rapid translocation from intracellular GLUT4 storage vesicles to the plasma membrane in response to decreased insulin levels in vivo, which promotes glucose uptake and maintains glycemic homeostasis, merits further investigation.

#### 4.3.2 Asparagine‐linked glycosylation protein 5 homolog (ALG5)

N‐glycosylation is a complex process that encompasses two distinct stages. In the first stage, precursor pyrophosphate polysaccharides, known as polysaccharides or dolichol‐linked oligosaccharides (DLO), are synthesized on the endoplasmic reticulum membrane. These polysaccharides, composed of fourteen‐carbon oligosaccharides (Glc3Man9GlcNAc), are subsequently transferred in their entirety to proteins by oligosaccharyltransferase (OST) on the luminal side of the endoplasmic reticulum.^122^, ^123^ This process is facilitated by a series of enzymatic reactions and glycosyltransferases.

On the luminal side of the endoplasmic reticulum membrane, DLO biosynthesis utilizes sugars conjugated to dolichol phosphate (dolichylphosphate mannose (Dol‐P‐Man) and dolichol phosphate glucose (Dol‐P‐Glc)) as the sugar‐donor substrates. Early studies identified transmembrane proteins encoded by the ALG5 gene that possess Dol‐P‐Glc synthase activity.^124^ It was later revealed that Dol‐P‐Glc is formed through the addition of glucose from uridine diphosphate‐glucose (UDP‐Glc) by ALG5 to Dol‐P.^125^ Notably, disruptions in ALG5 function would lead to the accumulation of incompletely glycosylated proteins.^126^ Similar findings were observed in Drosophila, where mutations in the ALG5 gene resulted in a reduced number of glycosylated and N‐glycosylated proteins.^127^

Thus, ALG5 encodes a transmembrane‐bound enzyme in the polyterpene glycol cycle of the endoplasmic reticulum, specifically acting as a polyterpene glycol phosphate β‐glucosyltransferase. It catalyzes the synthesis of Dol‐P‐Glc by adding three glucose residues and plays a crucial role in N‐glycosylation modifications. Unfortunately, the limited number of reports on ALG5 underscores the need for additional functional studies to address the knowledge gap surrounding this gene.

#### 4.3.3 Staurosporine and temperature sensitive 3A (STT3A)

The pivotal stage in the N‐glycosylation process involves the transfer of the glycan (Glc3Man9GlcNAc2) by OST, a complex protein assembly comprising a catalytic subunit, either STT3A or STT3B, along with six shared subunits and specific auxiliary subunits.^128^, ^129^ STT3A is linked to protein translocation channels and operates in a cotranslational manner, while sites lacking STT3A can undergo posttranslational glycosylation through STT3B.^130^ Depletion of STT3A triggers endoplasmic reticulum stress and induces the unfolded protein response, while STT3B compensates by hyperglycosylating associated proteins and facilitating their degradation via the lysosomal pathway.^130^ Wang et al. demonstrated that STT3A's abnormal expression may contribute to the heightened expression and membrane transport ratio of GLUT1, forming an STT3A‐GLUT1‐glucose uptake axis.^131^

To summarize, STT3A serves as the active center of OST isoforms and plays a crucial role in N‐glycosylation. Meanwhile, the glycoprotein GLUT undergoes N‐glycosylation in the rough endoplasmic reticulum, enabling its translocation from the cytoplasm to the cell membrane for optimal glucose transporter capacity. When the expression of STT3A is reduced, it results in abnormal N‐linked glycosylation, leading to decreased GLUT expression and a reduced rate of membrane translocation. Consequently, this impairment affects cellular glucose uptake. Therefore, conducting comprehensive research on STT3A could greatly contribute to future therapies targeting N‐glycosylation inhibition.

## 5 SIGNALING OF GLUCOSE METABOLISM IN AGING HIPPOCAMPUS

**Figure 2:**
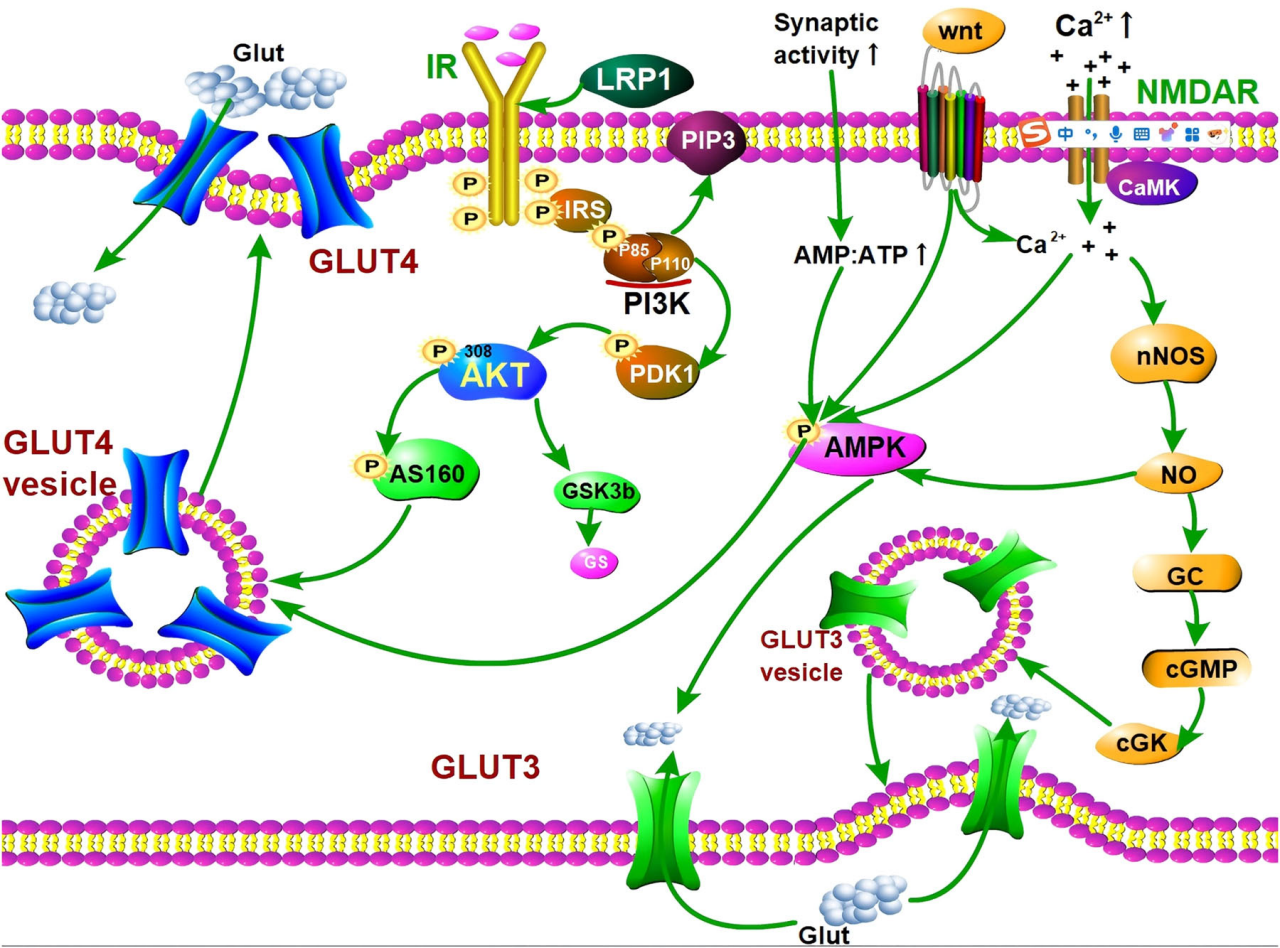
The metabolic signaling pathway associated with neuronal GLUTs. The canonical signaling pathway of the IR initiates with the binding of insulin. This prompts the phosphorylation of the insulin receptor substrate's phosphotyrosine binding (PTB) domain, which subsequently interacts and activates the downstream signaling molecule PI3K, leading to the phosphorylation of AKT, thereby phosphorylating AS160, promoting the translocation of GLUT4 to the cellular membrane. Elevated levels of AMP:ATP resulting from presynaptic energy stress, as well as increased neuronal activity leading to heightened Ca^2+^ levels, activate the local AMPK signaling pathway. Consequently, AMPK signaling facilitates the aggregation of GLUT4 at the presynaptic membrane surface by promoting its translocation. LRP1 interacts with the IR β‐subunit and modulates insulin transport and downstream neuronal effects mediated by the IR. This, influences GLUT4 translocation during signaling. Overactivation of NMDAR in response to heightened Ca^2+^ influx leads to the activation of nNOS, resulting in increased intracellular NO levels. This activates GC, leading to the production of cGMP, which subsequently activates cGK. This activation triggers the translocation of GLUT3 to the cell surface, facilitating glucose transport. Additionally, cGMP activation of cGKII stimulates AMPK activation, consequently enhancing GLUT3 activity. Activation of the Wnt/Ca^2+^ pathway promotes rapid Ca^2+^ movement, ultimately activating AMPK signaling and stimulating glucose uptake. AMPK, AMP‐activated protein kinase; CaMK, calmodulin‐dependent protein kinase; cGKII, cGMP‐dependent protein kinase II; cGMP, cyclic guanosine monophosphate; GC, guanylate cyclase; GLUT, glucose transporter; IR, insulin receptor; IRS, insulin receptor substrates; LRP, lipoprotein receptor‐related protein; NMDAR, NMDA receptors; nNOS, neuronal nitric oxide synthase; NO, nitric oxide; PDK1, phosphatidylinositol‐dependent protein kinase‐1; PI3K, phosphatidylinositol‐3 protein kinase; PIP3, 3,4,5‐phosphatidylinositol‐3,4,5‐trisphosphate. [Color figure can be viewed at wileyonlinelibrary.com]

The aforementioned analysis highlights the significance of GLUT1, GLUT3, GLUT4, IR, and IGF‐1R, as well as N‐glycosylation, in the context of glucose metabolism in the hippocampus of the brain. Their involvement appears to be particularly critical for maintaining the function, vulnerability, and plasticity of hippocampal neurons throughout the aging process. As a result, there has been considerable interest in unraveling the molecular mechanisms and signaling pathways that underlie the regulation of these processes to gain deeper insights into their intricate workings (Figure 2).

### 5.1 Insulin PI3K/AKT signaling pathways and glucose metabolism in the brain

The intricate process of glucose conversion within the human body is regulated by multifaceted mechanisms, among which insulin serves as a crucial regulatory pathway. Glucose uptake and metabolism are primarily regulated at the molecular level through the insulin signal transduction pathway. The phosphatidylinositol‐3 protein kinase (PI3K) pathway serves as the primary conduit for signal transduction.^132^

The canonical signaling pathway of the IR commences with the binding of insulin to its receptor. This engagement triggers a conformational alteration in the extracellular α‐subunit of the IR, culminating in the activation of the β‐subunit through the autophosphorylation of a specific tyrosine residue. Subsequently, the activated β‐subunit interacts with the IRS‐1/2, whereby the phosphorylated phosphotyrosine binding (PTB) domain of the IRS is recognized by the downstream signaling molecule PI3K. Subsequently, PI3K instigates the phosphorylation of phosphatidylinositol, generating 3,4,5‐phosphatidylinositol‐3,4,5‐trisphosphate (PIP3). PIP3 serves as the docking site for the serine/threonine protein kinase AKT and the phosphatidylinositol‐dependent protein kinase‐1 (PDK1). PDK1 phosphorylates AKT at threonine 308, while mechanistic target of rapamycin complex 2 (mTORC2), which is indirectly activated by PDK1, phosphorylates AKT at serine 473, thereby fully activating AKT. The fully activated AKT orchestrates a multitude of downstream signaling molecules.^133^

#### 5.1.1 PI3K/AKT/AS160/GLUT4

The initial and swift responder to AKT activation is AS160 (AKT substrate of 160 kDa), a Rab‐GTPase activating protein. In its unphosphorylated state, AS160 is localized to GSVs. However, upon phosphorylation by AKT, AS160 undergoes a conformational change that transforms Rab from its guanosine diphosphate (GDP)‐bound state to the active guanosine 5′‐triphosphate (GTP)‐bound form. This switch facilitates the translocation of the glucose transporter GLUT4 to the cell membrane, enabling glucose uptake.^134^ Insulin exerts a beneficial effect on brain energy regulation and function. Grillo et al.^135^ discovered that insulin administration into the lateral ventricle resulted in increased AKT phosphorylation and subsequent translocation of GLUT4 to the plasma membrane of the hippocampus, ultimately improving neuronal energy supply. Insulin prompts GLUT4 translocation to neuronal membranes via activation of the PI3K‐AKT signaling pathway within the brain, promoting glucose delivery to neurons for the maintenance of their structural and functional integrity during learning and memory processes.^136^ It is therefore essential to maintain normal insulin sensitivity. Cellular impairments in IR structure, function, and action lead to reduced sensitivity to receptor signaling and inadequate glucose utilization by cells, resulting in insulin resistance.^137^ Insulin resistance hampers GLUT4 translocation and reduces glucose uptake by cortical hippocampal neurons. This reduction in neuronal glucose uptake is often observed in neurodegenerative diseases.^138^, ^139^ It is important to note that simply increasing peripheral glucose concentration will not rectify cerebral energy deficits once glycolysis and neuronal function have been disrupted; in fact, it may exacerbate insulin resistance.^140^ As insulin and IGF1 receptor signaling diminish, varying degrees of disruption occur in neuronal function, synaptic plasticity, and cellular integrity.^136^, ^139^

#### 5.1.2 PI3K/AKT/GSK3β/GYS1

Phosphorylation of glycogen synthase kinase 3β (GSK3β), being a substrate of AKT, leads to its inhibition, resulting in increased glycogen synthase activity and enhanced glucose uptake by cells.^141^ Additionally, GSK‐3β has a negative feedback impact on the insulin signaling pathway.^142^ Notably, senescence is associated with heightened proinflammatory prostaglandin E2 (PGE2) signaling in myeloid cells, which shifts glucose metabolism towards the AKT‐GSK3β‐glycogen synthase 1 (GYS1) pathway, promoting glycogen production while reducing ATP synthesis.^143^ Glycogenolysis serves as an ATP source via glucose oxidation, temporarily sustaining neuronal signaling until brain energy failure, and facilitating the recruitment of GLUT4 proteins rapidly translocate from internal stores to presynaptic plasma membranes.^144^

### 5.2 The AMPK signaling and glucose metabolism

AMP‐activated protein kinase (AMPK) plays a pivotal role as a prominent detector of cellular energy stress. Its activation orchestrates the intricate regulation of both glycolysis and mitochondrial metabolism.^145^, ^146^ The synaptic activity imposes substantial energy stress on the presynaptic environment, characterized by an imbalance in ATP supply and heightened energy expenditure. As a consequence, the AMP:ATP ratio becomes elevated, serving as the impetus for the activation of local AMPK signaling through phosphorylation of the AMPK alpha subunit.^147^ Moreover, during intensified neuronal activity, augmented levels of mitochondrial Ca^2+^ play an instrumental role in activating local AMPK signaling via Ca^2+^/calmodulin‐dependent protein kinase (CaMKK), and potentially through the involvement of other Ca^2+^‐regulated protein kinases. Simultaneously, the aforementioned regulatory processes induce the phosphorylation of AS160 and TBC1 domain family member 1 (TBC1D1) at multiple phosphorylation sites, engendering the aggregation of glucose transporter GLUT4 to the surface of the presynaptic membrane. This mobilization of GLUT4 serves as an immediate response to sustained synaptic activity, facilitating glucose uptake and glycolysis.^148^ Intriguingly, the inhibition of GLUT4 was found to impede synaptic vesicle cycling during sustained action potential discharges.^84^ Studies reported the indispensable role of GLUT4 recruitment to the presynaptic plasma membrane in sustaining intense neuronal firing. Furthermore, they demonstrated that the blockade of GLUT4 in the rat hippocampus leads to impairments in memory formation and consolidation.^90^, ^149^ While the AMPK‐GLUT4 hypothesis holds promise, further investigations are imperative to elucidate the underlying mechanisms governing GLUT4 recruitment to the presynaptic plasma membrane and how its upregulation during prolonged excitation influences glycolysis and downstream oxidative pathways.

### 5.3 The Ca^2+^/NMDAR‐nNOS/NO/GCs signaling and glucose metabolism

The N‐methyl‐d‐aspartate receptor (NMDAR), plays a pivotal role in neuritogenesis, synaptic maturation, plasticity, and cognition.^150^, ^151^ Upon activation by the neurotransmitter glutamate, NMDAR opens the Ca^2+^ channel, leading to an increased influx of Ca^2+^ into the neurons. To maintain energy homeostasis during synaptic transmission, the over‐activation of NMDAR under intensified neuronal activity triggers the activation of neuronal nitric oxide synthase (nNOS), thereby elevating intracellular levels of nitric oxide (NO). One of the primary targets of NO in neuronal cells is guanylate cyclase (GC), which generates cyclic guanosine monophosphate (cGMP) to activate cGMP‐dependent protein kinase (cGK). Subsequently, this activation triggers the translocation of GLUT3 to the cell surface, facilitating glucose transport.^75^, ^152^, ^153^ Additionally, cGMP‐dependent protein kinase II (cGKII) contributes to the surface expression of AMPA receptors at extrasynaptic sites, activating AMPK, and enhancing GLUT3 activity.^154^ Along similar lines, under hypoxic conditions, NO has been shown to enhance GLUT3 activity through the activation of AMPK.^155^ Neuronal membrane depolarization triggers the fusion of GLUT3‐containing vesicles with the plasma membrane, resulting in a notable surge in glucose uptake.^156^ Furthermore, insulin has been shown to enhance KCl‐induced glucose uptake in Ca^2+^‐dependent neurons, accompanied by an augmented presence of GLUT3 on the outer surface of the plasma membrane. This suggests that insulin promotes GLUT3 translocation and subsequent glucose transport.^157^ While the Ca^2+^/NMDAR‐induced increase in surface GLUT3 is not the primary pathway for neuronal glucose metabolism in the brain, it still plays a vital role in preserving glucose homeostasis during neuronal transmission.

### 5.4 The Wnt signaling and glucose metabolism

Extensive research has demonstrated the pivotal role of the Wnt signaling pathway in physiological processes within the CNS. These processes include synaptogenesis, synaptic vesicle cycling, neurotransmitter receptor transport, and most notably, synaptic remodeling, vesicle cycling, and synaptic transmission in hippocampal neurons.^158^, ^159^, ^160^ Notably, studies have revealed that the ATP‐dependent activation of key proteins in the Wnt pathway at synapses plays a crucial role in regulating energy metabolism within the brain.^161^ Activation of the Wnt/Ca^2+^ pathway in the brain facilitates rapid Ca^2+^ movement, subsequently activating multiple Ca^2+^‐dependent enzymes downstream. The modulation of Ca^2+^ levels significantly impacts glucose metabolism in neurons and astrocytes.^162^ The role of Ca^2+^ in regulating glucose metabolism in neurons has been previously discussed. Additionally, Wnt family member 5A (Wnt5a), a member of the nonclassical Wnt family, regulates hexokinase activity and the rate of glycolysis, as well as glucose 6‐phosphate dehydrogenase (G6PDH) and pentose phosphate pathway (PPP) activity. These effects are mediated through NO‐related signaling downstream of Wnt5a ligand.^163^ Wnt5a thus regulates cellular glucose metabolism in neurons in an NO‐dependent manner. The effects of NO on glucose metabolism in neurons have been outlined earlier. Furthermore, nonclassical Wnt signaling activates AMPK signaling, promoting glucose uptake and oxidation.^164^, ^165^

### 5.5 LRP1 and glucose metabolism

Low‐density lipoprotein receptor‐related protein (LRP), a cell membrane protein, is categorized as an endocytic receptor possessing multifunctional scavenger and signaling properties. The study by Jedrychowski et al. suggested a necessity for LRP1 expression in the formation of functional GSVs.^166^ Subsequent in vitro and in vivo modeling investigations shed further light on the interactions between LRP1 and the IR β subunit within the brain. It plays a regulatory role in insulin transport in neurons by modulating the IR and its downstream signaling during GLUT4 translocation. These findings collectively indicate that LRP1 engages with the IR β subunits to regulate GLUT4 expression, ultimately leading to decreased insulin‐stimulated glucose uptake. The interaction between LRP1 and IR β subunits and their influence on GLUT levels establish a potential mechanism for the regulation of insulin signaling and glucose metabolism in the brain.^167^

### 5.6 N‐glycosylation and glucose metabolism

All members of the GLUT family possess a conserved N‐glycosylation site (Asn‐Xaa‐Thr/Ser) within the extracellular loop connecting transmembrane domains 1 and 2. Notably, a relevant research investigation focused on GLUT1 revealed the presence of a solitary N‐linked glycosylation site at asparagine 45 (N45).^168^ A lot of research corroborated the significance of N‐glycosylation in GLUT1, not only for its catalytic activity as a transporter protein but also for its proper subcellular localization.^169^, ^170^ In the nervous system, N‐glycans contribute to the enhancement of glycoprotein functionality through several mechanisms. They facilitate protein folding and transportation to the cell surface, promote protein stability by regulating uptake and recycling to the plasma membrane, and enhance protein activity by modifying biophysical properties.^171^ It is worth noting that the number of glycans and the specific glycan structure can vary among different proteins, thereby exerting distinct effects on protein function. Interestingly, among the GLUTs, only GLUT4 possesses two glycan‐binding sites, implying its potential divergence in glucose transport capabilities.

**Figure 3:**
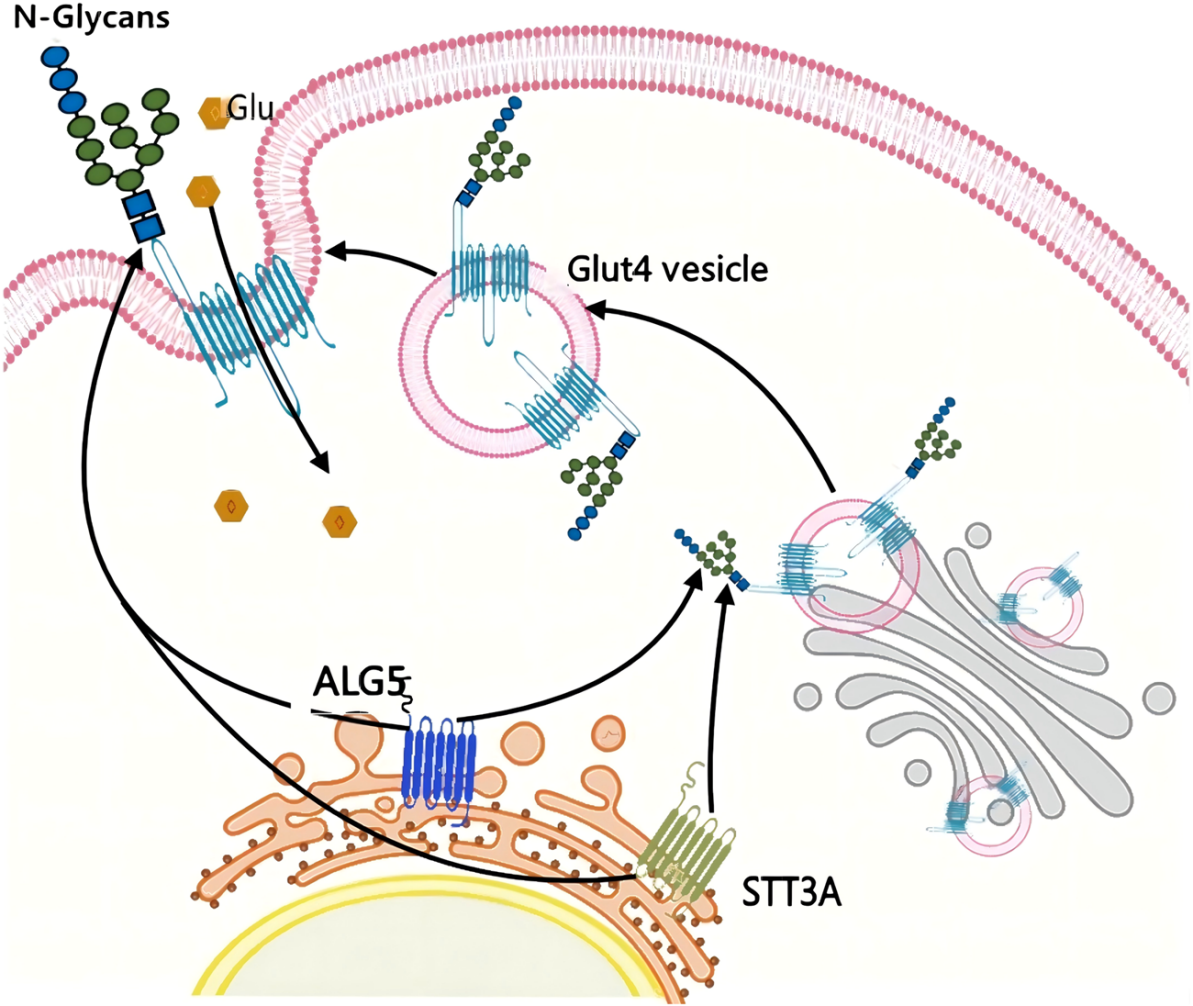
The regulation of GLUT4 by N‐glycans mediated through ALG5 and STT3A. In the physiological state, the majority of GLUT4, more than 90%, is localized in various compartments such as the Golgi apparatus, microsomes, tubular vesicles, and GLUT4 storage vesicles (GSVs). Upon stimulation by insulin, there is a rapid translocation of GLUT4 from GSVs to the plasma membrane system. Notably, the extracellular loop between domains 1 and 2 of the GLUT4 transmembrane structure contains N‐glycosylation sites, which can regulate the functionality of the channel. The presence of N‐glycans in this region plays a crucial role in facilitating the folding and transportation of glycoproteins to the cell surface, with STT3A and ALG5 involved in this process. This, in turn, impacts their stability and distribution on the cell surface, including their recycling at the plasma membrane, and alters their molecular properties. ALG5, asparagine‐linked glycosylation protein 5 homolog; Glut, glucose transporter; STT3A, staurosporine and temperature sensitive 3A. [Color figure can be viewed at wileyonlinelibrary.com]

The findings indicated that insulin treatment led to an elevation in the cell surface expression of wild‐type GLUT4, whereas the glycosylation mutant lost its responsiveness to insulin. This substantiated the notion that the N‐glycan structure plays a crucial role in the overall stability of newly synthesized GLUT4, contributing to quality control and intracellular transport.^172^ Another study performed multicolor imaging of the GLUT 4 translocation, proving N‐glycan‐deficient GLUT4 was transiently translocated to the cytosolic membrane upon insulin stimulation, followed by rapid internalization without retention. These findings strongly suggest that the N‐glycan structure is instrumental in maintaining the overall stability of newly synthesized GLUT4, and plays a role in its retention at the cell membrane.^173^ Taken together, these findings underline the critical nature of plasma membrane retention for GLUT4 in insulin signaling, with N‐glycosylation playing an essential role in maintaining its presence at the plasma membrane for effective glucose transport. Key components involved in this process include STT3A, which serves as the active center of the OST responsible for the central step in glycan transfer during N‐glycosylation. Additionally, ALG5 is the enzyme involved in catalyzing the sugar‐donor substrate Dol‐P during N‐glycosylation modification. Both STT3A and ALG5 are essential enzymes that can influence various aspects of GLUT4, including protein folding, stability, transport, and other related processes (Figure 3).

## 6 CONCLUSION AND PROSPECTS

Essentially, cognitive decline in elders stems from the irreversible loss and functional decline of neurons, with the hippocampus being particularly susceptible to age‐related neuronal deterioration. This region plays a crucial role in the transition from normal aging to neurodegenerative diseases. Glucose acts as the primary fuel for the brain, and a constant provision of glucose to the hippocampus is essential for memory and cognitive functions. The transport of glucose across the BBB into astrocytes or intercellular fluid within the brain is facilitated by GLUT1. GLUT3, on the other hand, is primarily responsible for ensuring a stable glucose supply to neurons, particularly in the hippocampus. It is closely associated with both neurodevelopmental and neurodegenerative processes. GLUT4, an insulin‐dependent glucose transporter, is predominantly found in the hippocampus. It potentially acts as a “reserve” channel to supply glucose to hippocampal neurons, thus impacting their function, metabolism, and aging. N‐glycosylation, a process where glucose molecules are attached to glycoproteins, enables the glucose transport capacity of GLUT. The initial step of N‐glycosylation requires Dol‐P‐linked glycans (Dol‐P‐Man and Dol‐P‐Glc) as glucose‐donor substrates. Dol‐P‐Glc, synthesized from UDP‐Glc via ALG5, is a crucial glucose donor incorporated into the glycosylation process. Therefore, ALG5 and STT3A, as key molecules involved in N‐glycosylation, play significant roles in regulating the expression of GLUT, membrane transport rates, and glucose homeostasis, thereby influencing cellular glucose uptake. The uptake of glucose by hippocampal neurons heavily relies on insulin levels. Fluctuations of glucose levels make the hippocampus particularly sensitive to insulin deficiency. Aging neurons are accompanied by an increase in insulin resistance and a decrease in sensitivity to IR, such as IR and IGF‐1R, resulting in inefficient glucose utilization. Consequently, neuronal function, synaptic plasticity, and cellular integrity are compromised to varying extents, leading to ineffective glucose utilization by hippocampal neurons. Several signaling pathways in the brain, notably the insulin PI3K/AKT/AS160 pathway, greatly impact glucose metabolism. This pathway predominantly regulates the translocation of GLUT4, which possesses two unique glycosylation sites. When insulin concentrations decrease in vivo, GLUT4 rapidly relocates from GSVs to the plasma membrane, promoting glucose uptake and maintaining blood glucose homeostasis. They pose a significant research prospects for the future for understanding the influence of glucose metabolism on hippocampal neuron aging and the specific mechanisms involved.

## AUTHOR CONTRIBUTIONS

Gaohong Zhu contributed to the central idea and the revision. Rui He completed the literature search, collation, and paper writing. Fuxing Zhao, Zhiyu Yang, Tingting Wang, Yaohui Zhang, and Jinglan Quan completed the literature search.

## CONFLICT OF INTEREST STATEMENT

The authors declare no conflicts of interest.

## ETHICS STATEMENT

Not applicable.

## Data Availability

Not applicable as no new data were generated in this review.
